# Actomyosin contractility scales with myoblast elongation and enhances differentiation through YAP nuclear export

**DOI:** 10.1038/s41598-019-52129-1

**Published:** 2019-10-29

**Authors:** Céline Bruyère, Marie Versaevel, Danahe Mohammed, Laura Alaimo, Marine Luciano, Eléonore Vercruysse, Sylvain Gabriele

**Affiliations:** University of Mons, Laboratory for Complex Fluids and Interfaces, Mechanobiology and Soft Matter group, Research Institute for Biosciences, Place du Parc, 20, B-7000 Mons, Belgium

**Keywords:** Biophysics, Materials science

## Abstract

Skeletal muscle fibers are formed by the fusion of mononucleated myoblasts into long linear myotubes, which differentiate and reorganize into multinucleated myofibers that assemble in bundles to form skeletal muscles. This fundamental process requires the elongation of myoblasts into a bipolar shape, although a complete understanding of the mechanisms governing skeletal muscle fusion is lacking. To address this question, we consider cell aspect ratio, actomyosin contractility and the Hippo pathway member YAP as potential regulators of the fusion of myoblasts into myotubes. Using fibronectin micropatterns of different geometries and traction force microscopy, we investigated how myoblast elongation affects actomyosin contractility. Our findings indicate that cell elongation enhances actomyosin contractility in myoblasts, which regulate their actin network to their spreading area. Interestingly, we found that the contractility of cell pairs increased after their fusion and raise on elongated morphologies. Furthermore, our findings indicate that myoblast elongation modulates nuclear orientation and triggers cytoplasmic localization of YAP, increasing evidence that YAP is a key regulator of mechanotransduction in myoblasts. Taken together, our findings support a mechanical model where actomyosin contractility scales with myoblast elongation and enhances the differentiation of myoblasts into myotubes through YAP nuclear export.

## Introduction

Skeletal muscles are the most abundant muscles of the human body and ensure the maintenance and movement of the body. Disruption of the contractile activity of skeletal muscles is associated with a progressive muscle degeneration and weakness, as observed for instance in sarcopenia^1^ and Duchenne muscular dystrophy (DMD)^2,3^. Skeletal muscles are formed from myoblasts, which are mononucleated cells, that fuse together to form long multinucleated cells, called myotubes. Myotubes differentiate and then reorganize into multinucleated myofibers that assemble in bundles to form skeletal muscles^4^. In the early stages of muscle differentiation *in vitro*, myoblasts proliferate, recognize each other, align, and eventually form fusion pores and fuse in multinucleated myotubes^5–7^.

Interestingly, myoblast fusion is tightly controlled by spatial cues. Indeed, myoblasts usually fuse into long linear myotubes that require the elongation of myoblasts when they align and fuse. The seminal work of Clark and coworkers provided evidence of the importance of myoblast elongation by studying myoblast fusion on laminin tracks of different widths^8^. They found that although the number of myoblasts increased with the line width, the diameter of myotubes formed remained constant. Interestingly, time-lapse imaging of fusing myoblasts *in vitro* has reported end-to-end fusion^9^ and emphasized the importance of both side-to-side and end-to-end interactions^10^. Elongation of myoblasts is likely to be the result of a deep reorganization of the networks of actin filaments and microtubules that must align parallel to the long axis of the cell^11^. Furthermore, it was previously described that bundles of actin stress fibers can be found in migrating myoblasts, but not in aligned cells where they are mainly found at the cell periphery, suggesting that the remodeling of the actin cytoskeleton is essential for myoblast fusion^12,13^.

It has been shown that the formation of an actin wall structure can temporally restricts the initiation of membrane fusion until myoblasts have aligned and elongated to become bipolar cells^14^. Moreover, inhibition of non-muscle myosin IIA motor activity prevents formation of this cortical actin wall, as well as appearance of vesicles that need to pair across the aligned myoblasts. More recent studies in cultured cells suggest that RhoA activity must be tightly regulated in a finely coordinated time-dependent manner to ensure appropriate skeletal muscle formation^15^. The modulation of RhoA activity in myoblasts was found to be essential for subsequent cell cycle withdrawal, expression of skeletal muscle differentiation genes, and myotube fusion. In addition, it has been suggested that myoblast fusion is associated with a modification of the balance between cell-substrate and cell-cell adhesions. Indeed, fusing myoblasts tend to become less attached to the culture substrate, whereas cell-cell interactions become more important^16^.

Although a complete understanding of the mechanisms governing skeletal muscle fusion is lacking, it is clear that mechanical forces play an integral role in this biological process. For instance, desmin mutation were found to alter traction forces in C2C12 cells, that lack organized sarcomeres^17,18^. It is therefore reasonable to consider cell aspect ratio, spatial organization of the actin cytoskeleton and traction forces as potential regulators of the fusion of myoblasts into myotubes. To address this issue, we cultured single myoblasts obtained from an immortalized mouse cell line (C2C12) on hydroxy-polyacrylamide (hydroxy-PAAm) hydrogels^19,20^ with stiffness typical of normal muscle (~12 kPa)^21,22^. C2C12 myoblasts were cultured on hydroxy-PAAm hydrogels functionalized with circular (CSI = 1), square (CSI = 0.79), triangular (CSI = 0.60) and rectangular micropatterns (CSI = 0.50 and 0.34 for 1:4 and 1:7 aspect ratios, respectively) of fibronectin (FN). These different geometries of micropatterns allowed to standardize *in vitro* the myoblast shape over a wide range and to control their spreading area. By combining confocal microscopy imaging with traction force microscopy (TFM), we asked whether cell shape regulates the cytoskeletal organization of myoblasts and their contractile forces exerted on the substrate. Knowing that traction forces in smooth muscle cells varies with cell spreading^23,24^, we imposed a constant micropattern area of 1600 µm^2^ to standardize the spreading of myoblasts for studying in a robust way the relation between cell shape, traction forces and fusion. To determine the role of the actomyosin network in cell fusion and differentiation, we used Latrunculin B (LatB) to disrupt actin filaments and Blebbistatin (Bleb) to inhibit non-muscle myosin II of both individual micropatterned myoblasts and dense cultures of myoblasts. Then we observed pairs of C2C12 myoblasts adhered to micropatterns of different CSI to investigate whether the contractile forces of a fusing cell pair are modulated by the cell shape.

Finally, we investigated the role of the transcriptional co-activator Yes-associated protein (YAP) during the fusion and differentiation processes of myoblasts. YAP is a transcriptional coactivator downstream of the Hippo pathway that regulates many cellular functions, such as proliferation, migration, differentiation, and apoptosis^25^. The Hippo pathway member YAP has been shown to be involved in skeletal muscle development and regeneration^26–28^, to contribute to the regulation of activation, proliferation and differentiation of satellite cells^29^ and to modulate myogenesis and muscle regeneration^30^, whereas abnormal YAP activity has been reported in muscular dystrophy and rhabdomyosarcoma^31^. Precise mechanisms by which YAP is regulated by mechanical cues in myoblasts are still unknown, even if cytoskeletal tension has been suggested as an important player of YAP-mediated mechanostransduction. To address this question, we investigated the interplay between myoblast elongation, YAP nuclear export and differentiation rate into myotubes.

## Results

### Myoblasts adopt an elongated shape to differentiate and regulate their actin network to the spreading area

**Figure 1:**
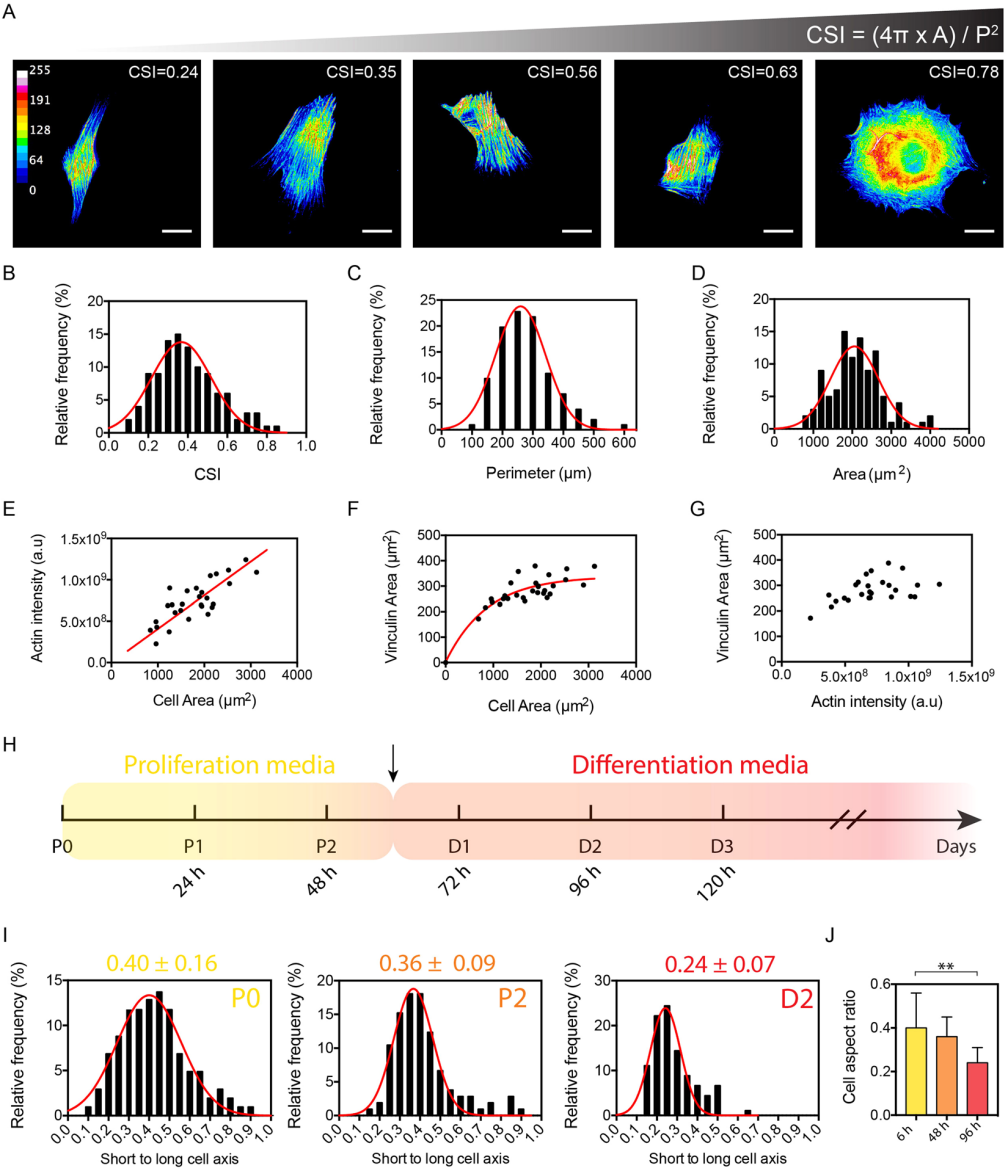
Myoblasts adopt an elongated shape to differentiate and regulate their actin network to the spreading area. (**A**) Color-coded epifluorescence images of typical C2C12 individual myoblasts exhibiting various Cell Shape Indexes (CSI) *in vitro*. Cells were stained with phalloidin for actin. Scale bars are 20 µm. The morphological analysis of (**B**) the CSI (R^2^ = 0.80), (**C**) the cell perimeter (R^2^ = 0.97) and (**D**) the cell area (R^2^ = 0.82) indicate that C2C12 cells cultured *in vitro* exhibited a mean area of 2057 ± 618 µm^2^ and a mean CSI of 0.37 ± 0.15 (n = 101 for B, C and D). Red curves are Gaussian fits. (**E**) Linear evolution of the fluorescence intensity of actin as function of the cell area (R^2^ = 0.748, n = 28). Evolution of the total vinculin area as function of (**F**) the cell area (R^2^ = 0.783, n = 28) and (**G**) and the intensity of F-actin (n = 28). (**H**) Timeline of proliferation (in yellow) and differentiation (in red) of C2C12 myoblasts *in vitro*. The black arrow indicates the replacement of proliferation media with differentiation media. (**I,J**) Aspect ratio of myoblasts at P0, P2 (proliferation stages, mean = 0.40 ± 0.16 at P0, n = 150 for both) and D2 (differentiation stage, mean = 0.24 ± 0.07, n = 100).

To assess the relationship between cell shape, actin network and focal adhesions, we cultured C2C12 myoblasts on a homogeneous layer of fibronectin (FN). After 24 hours in culture, myoblasts were fixed and stained for actin to determine their morphological parameters (Fig. 1A). FN-coated substrates allowed the establishment of specific cell-substrate interactions by recruiting specific transmembrane integrins. Indeed, several α integrin subunits that combine with β1 subunit are expressed during skeletal myogenesis, being important in myogenic cell migration, myoblast fusion, muscle fiber maturation or in maintenance of muscle integrity^32,33^. Additional experiments performed on laminin(LAM)-coated substrates indicated that both morphological parameters (cell shape index, cell perimeter and cell area, Supplementary Fig. 1A) and cell-substrate interactions (number and area of focal adhesions, Supplementary Fig. 1B) were statistically similar on FN and LAM, validating the FN coating for studying *in vitro* the morphologies of C2C12 myoblasts during the fusion/differentiation process.

We quantified the cell shape index (CSI) that characterized the morphology of myoblasts by giving information on the cellular elongation. Rounded cells have a CSI close to 1, while elongated cells have a CSI close to 0. We found CSI ranging from 0.1 to 0.8 with a mean value of 0.37 ± 0.15 (n = 101), suggesting a large variability in cell morphologies (Fig. 1B). Myoblasts exhibited a mean perimeter of 259 ± 82 µm (Fig. 1C, n = 101) and wide range of spreading areas (from ~1000 to ~4000 µm^2^), with a mean value of 2057 ± 618 μm^2^ (Fig. 1D, n = 101). These findings obtained on C2C12 myoblasts were strengthened by performing additional morphological measurements (CSI, cell perimeter and cell area) on primary human myoblasts (16UBic, Supplementary Fig. 2A) that come from the biceps of a patient unaffected by the FSHD (i.e. who have been confirmed to lack a 4qA deletion). As shown in Supplementary Fig. 2B–D, our results demonstrate that the CSI of 16UBic myoblasts ranged from 0.14 to 0.86 with a mean value of 0.44 ± 0.17 (n = 90), suggesting a large variability in cell morphologies for human myoblasts, as observed for C2C12 cells. Taken together, our results demonstrate that the variability of cell morphologies is not dependent on the cell type or any artefact of the cell culture.

We next studied the distribution of actin filaments in a population of C2C12 myoblasts for understanding whether the variability spreading areas can modulate the actin cytoskeleton. Our findings showed that the total fluorescence intensity of the F-actin network was linearly related to the cellular area (Fig. 1E, n = 31, R^2^ = 0.748), suggesting that the larger the myoblast spread, the more actin filaments are formed. As shown in Supplementary Fig. 3, we next characterized the distribution of vinculin-contained adhesions to determine how variations of cell shape affect cell-substrate interactions in C2C12 myoblasts. We found that the total area of cell-substrate adhesions per cell increased with cell area (Fig. 1F, n = 31, R^2^ = 0.783) and with the fluorescence intensity of F-actin (Fig. 1G). Our results indicate that C2C12 myoblasts assume a variety of cell shapes and spreading areas, but in turn adapt both the amount of actin filaments and vinculin adhesions to their spreading. To gain more insight into the role of cell shape for myoblast differentiation, we next consider the evolution of the cell aspect ratio during proliferation (P0 at 6 hours and P2 at 48 hours) and differentiation (D2 at 96 hours) stages (Fig. 1H). As shown in Fig. 1I,J, our findings show that myoblasts increased their aspect ratio from 0.40 ± 0.16 (P0) to 0.36 ± 0.09 (P2) and 0.24 ± 0.07 (D2), suggesting that myoblasts elongate significantly and adopt an aspect ratio of 1:4 to differentiate. In addition, our results indicated that the actin stress fibers were highly oriented at D2 (Supplementary Fig. 4A,B), corresponding also to significant nuclear elongations (Supplementary Fig. 4C). Taken together, our findings show that the F-actin network is modulated by modifications of the myoblast morphology and indicate that myoblasts differentiation required cellular elongation up to an aspect ratio of 1:4.

### The spatial organization of the actin network and the nucleus is directed by the myoblast morphology

**Figure 2:**
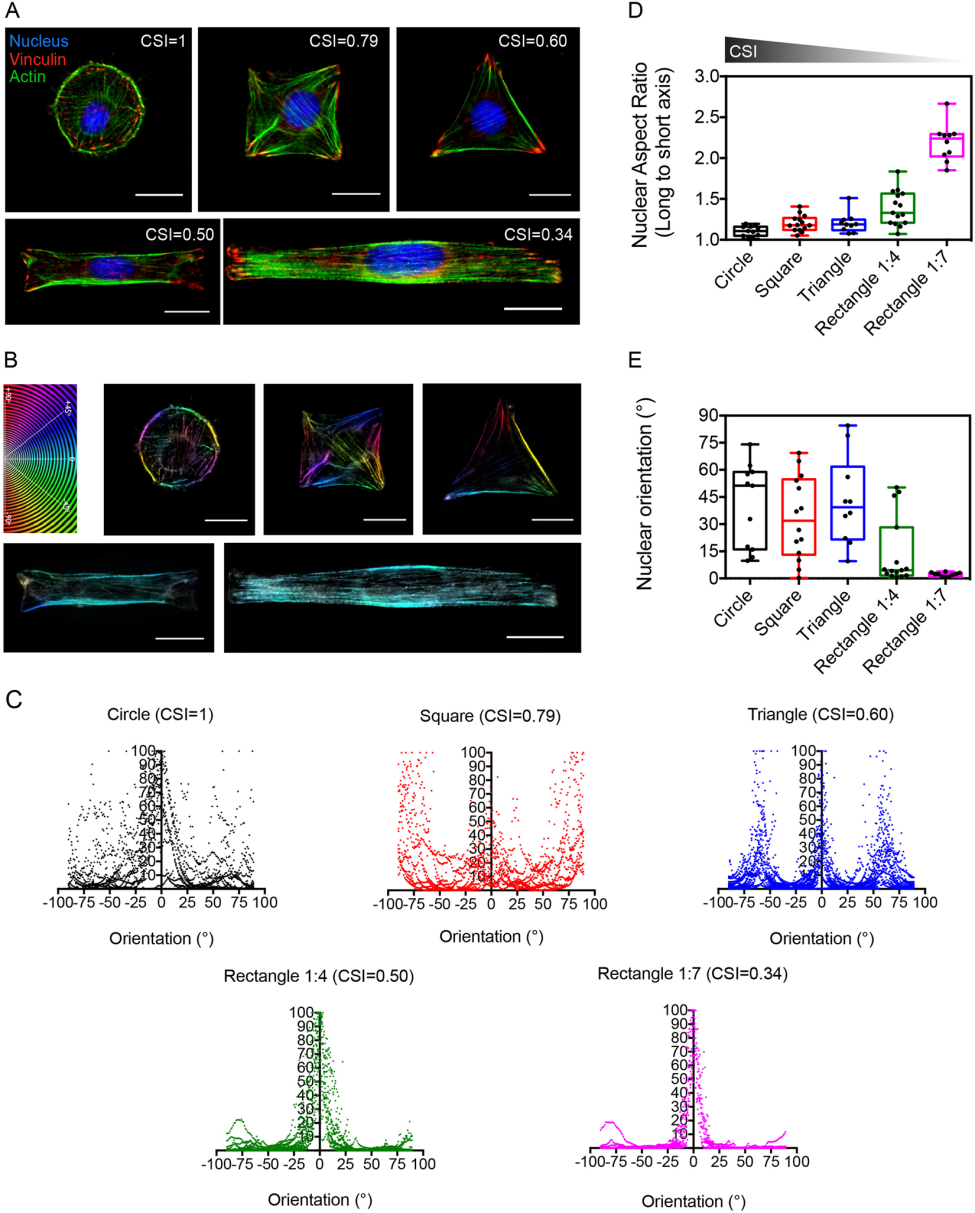
The spatial organization of the actin network and the nucleus is directed by the myoblast morphology. (**A**) Epifluorescence images of C2C12 cells grown on fibronectin (FN) micropatterns of 1600 µm^2^ and immunostained for F-actin (green), nuclei (blue) and vinculin (red). Circular, squared, triangular and rectangular (1:4 and 1:7 aspect ratios) micropatterns correspond to CSI = 1, 0.79, 0.60, 0.50 and 0.34, respectively. Scale bars are 20 µm. (**B**) Typical examples of the spatial organization of actin filaments in micropatterned C2C12 cells which are color-coded according to their orientations. Scale bars are 20 µm. (**C**) Orientation of the actin network in rounded (n = 12 in black), squared (n = 11 in red), triangular (n = 19 in blue) and 1:4 (n = 18 in green) or 1:7 (n = 11 in pink) rectangular micropatterned myoblasts. Evolution of (**D**) the nuclear aspect ratio and (**E**) the nuclear orientation for different geometries of myoblasts (10 ≤ n ≤ 15 for each CSI).

We controlled the intrinsic variability in myoblast morphologies by using adhesive micropatterns to standardize their spreading areas and control their shapes. By using a microcontact printing technique^20^, we created adhesive micropatterns of fibronectin (FN) with a constant area of 1600 µm^2^ and different geometries. We used rounded (CSI = 1), squared (CSI = 0.79), triangular (CSI = 0.60) and different aspect ratios of rectangular (CSI = 0.50 and 0.34 for 1:4 and 1:7 aspect ratios, respectively) FN micropatterns to culture individual myoblasts (Fig. 2A). These different geometries of micropatterns allowed to standardize *in vitro* the myoblast shape over a wide range and to control their spreading area.

To quantitatively determine to role of the cell geometry on the spatial organization of the actin cytoskeleton, we determined the orientation of the actin filaments for each cell shape^34,35^. Actin filaments stained with phalloidin were color-coded as a function of their orientation with a color gradient ranging from light blue when actin filaments were organized parallel (0°) to the horizontal axis and red (+90°) or orange (−90°) for those organized perpendicularly to the horizontal axis (Fig. 2B). We observed that actin filaments in rounded cells were distributed randomly, with orientations ranging from −90° to +90°. Squared cells exhibited actin filaments organized in three main angular domains: 0° to 25°, 50° to 90° and −50° to −90°, whereas triangular cells showed actin filaments mainly organized according to the three sides of the pattern at 0°, 60° and −60°. Interestingly, we found that actin filaments were highly oriented parallel to the long cell axis (0°) for rectangular cells of 1:4 (CSI = 0.50) and 1:7 (CSI = 0.34) aspect ratios. The quantification of vinculin-containing adhesions indicated no statistical differences of adhesion areas between cell morphologies (Supplementary Fig. 5A–C), whereas the orientation of focal adhesions was modulated by cell shape (Supplementary Fig. 5D,E), as observed for actin filaments. Our findings show that both 1:4 and 1:7 rectangular myoblasts allow a stronger organization of actin cytoskeleton (Fig. 2C) and focal adhesions, suggesting that myoblast elongation leads to the formation of contractile dipoles.

Considering the role of the cell nucleus in differentiation of myoblasts in myotubes, we next investigated whether changes in cell shape and cytoskeleton architecture can modulate the shape and orientation of the nucleus^36^. We found that the nuclear aspect ratio increased significantly with the lowering of the CSI (Fig. 2D). Indeed, rounded cells (CSI = 1) on circular micropatterns showed a rounded nucleus characterized by an average aspect ratio of 1.11 ± 0.06, whereas rectangular cells with an aspect ratio of 1:4 (CSI = 0.50) and 1:7 (CSI = 0.34) exhibited large nuclear deformations with aspect ratios of 1.39 ± 0.21 and 2.19 ± 0.23, respectively. We also noticed that the orientation of the nucleus was modulated by the cell morphology (Fig. 2E). We found that the orientation of the nucleus in rounded, squared and triangular cells spanned a wide range of angles (from ~15° to ~60°), with an average orientation of ~40° (circular, n = 11), ~33° (square, n = 14) and ~43° (triangle, n = 10) relative to the horizontal axis. For rectangular cells, our results indicated that the nucleus was oriented parallel to the cell axis with an average angle of ~14° (1:4, n = 15) and ~2° (1:7, n = 10).

### Cell elongation enhances myoblast contractility

**Figure 3:**
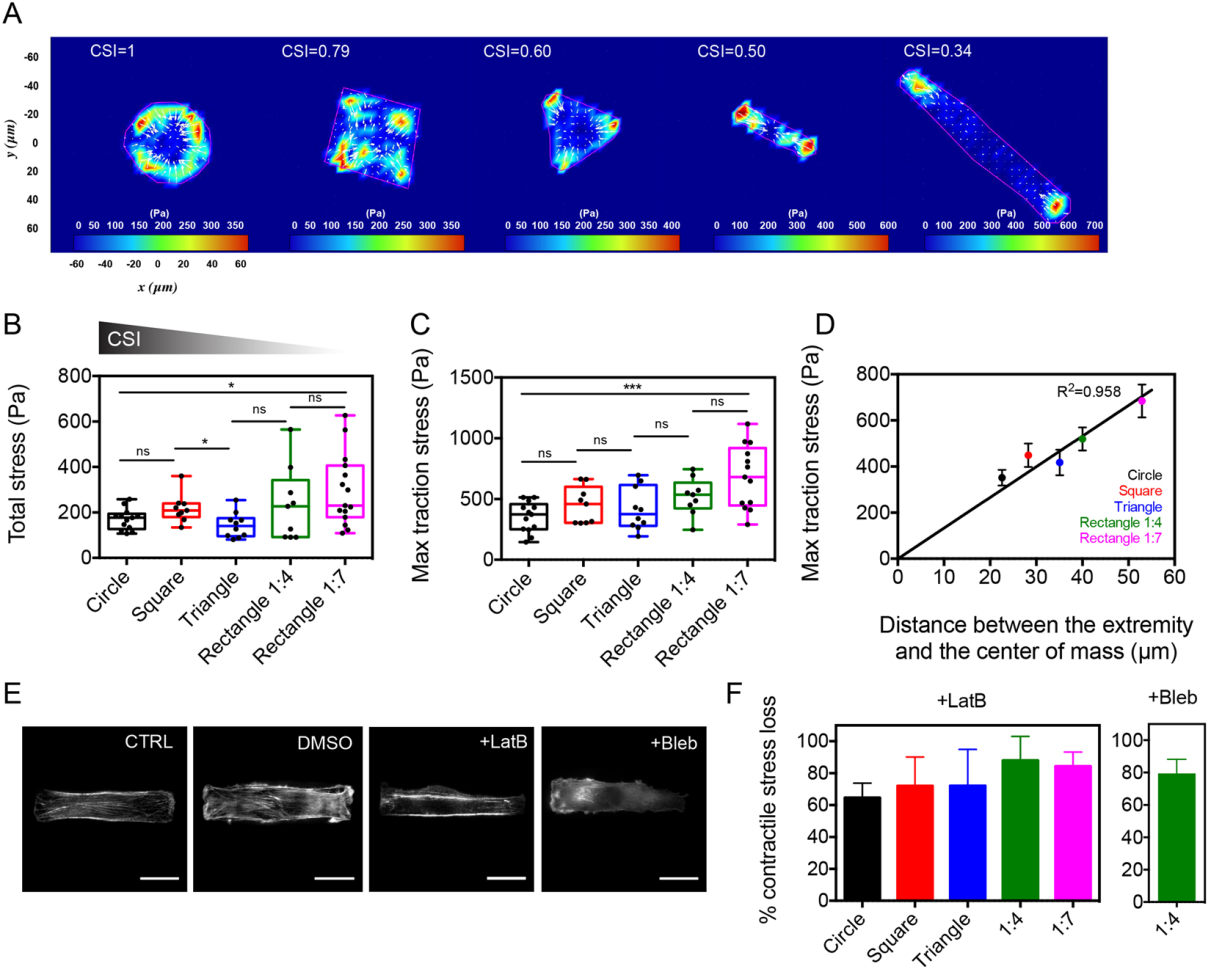
Actomyosin contractility is enhanced in elongated myoblasts. (**A**) Typical stress obtained by traction force microscopy (TFM) for five different myoblast morphologies (from CSI = 1 to CSI = 0.34). (**B**) Evolution of the total stress for different myoblast morphologies (n = 13 for CSI = 1, n = 9 for CSI = 0.79, n = 10 for CSI = 0.60, n = 9 for CSI = 0.50 and n = 13 for CSI = 0.34). Evolution of the maximal traction stress for (**C**) different myoblast morphologies and (**D**) as a function of the distance between the cell extremity and the center of mass. The black line is a linear fit (R^2^ = 0.958, 9 ≤ n ≤ 13 for each shape). (**E**) Typical immunofluorescence images of a 1:4 rectangular myoblast treated with DMSO, LatB and Bleb. (**F**) Loss of contractile stress in different myoblast morphologies in response to LatB and Bleb. For LatB-treated cells: n = 10 for CSI = 1, n = 8 for CSI = 0.79, n = 5 for CSI = 0.60, n = 10 for CSI = 0.50 and n = 6 for CSI = 0.34. For Bleb-treated cells: n = 8 for CSI = 0.50. Scale bars are 20 µm. *p < 0.05, **p < 0.01, ***p < 0.001 and n.s. not significant.

The next question we addressed was how changes in cell shape affect myoblast contractility. To answer this question, we used traction force microscopy (TFM) to determine the traction stresses exerted by micropatterned myoblasts. As shown in Fig. 3A we observed that the maximal stresses were exerted at the extremities of the micropatterned myoblasts, regardless the cell shape. As observed previously on other cell types^37^, the contractile stress accumulated preferentially in the corners (squares and triangles) or at both extremities (1:4 and 1:7 rectangles) of micropatterned myoblasts. We quantified the total stress exerted by individual micropatterned myoblasts to determine whether the cell morphology modulates the cell contractility. As shown in Fig. 3B, rounded cells (CSI = 1) exerted a total stress of 170.7 ± 46.4 N/m^2^, while rectangular cells (CSI = 0.34, aspect ratio 1:7) exerted a larger total amount of stress (295.9 ± 156.8 N/m^2^), suggesting that elongated cell shapes lead to increased traction stresses. Furthermore, we found that rectangular shaped cells (CSI = 0.34, aspect ratio 1:7) exhibited the larger maximal stress value (684.3 ± 257.8 Pa, Fig. 3C), while rounded myoblasts showed the lowest maximal stress value (351.5 ± 123.6 Pa). Considering that the contractile stresses were exerted at the cell periphery and that lowering the CSI leads to increased traction stresses, we plotted the maximal stress value as a function of the distance from the center of mass to the extremity of the cell (Fig. 3D), which represents 22.6 µm (CSI = 1), 28.28 µm (CSI = 0.79), 33.98 µm (CSI = 0.60), 41.23 µm (CSI = 0.50) and 53.45 µm (CSI = 0.34). As shown in Fig. 3D, we found that the maximal contractile stress increased linearly with the distance from the centroid to the extremity of the cell (R^2^ = 0.958, n = 65), suggesting that elongated morphologies of myoblasts exert more tractions forces.

In order to determine the contribution of the actomyosin network in the establishment of contractile forces in myoblasts, C2C12 cells were treated with the G-actin sequestering drug Latrunculin B (LatB) and with the myosin II inhibitor Blebbistatin (Bleb). Immunostained myoblasts with phalloidin indicated that LatB and Bleb-treated cells exhibited a diffuse actin network (Fig. 3E), demonstrating that both drugs affected significantly the organization of F-actin. We used TFM on micropatterned myoblasts treated with both drugs to determine the role of the actomyosin network in the establishment of contractile forces in myoblasts of different geometries. Our findings indicate that LatB treatment induced a significant loss of contractility, which increased with the cell elongation. Indeed, rounded cells showed a loss of contractile stress of ~62%, whereas 1:4 and 1:7 rectangular cells treated with LatB were characterized by a loss of contractile stress of ~88% and ~86%, respectively (Fig. 3F). In addition, we found that the contractile stress of 1:4 rectangular cells treated with Bleb were characterized by a loss of ~80% of the contractile force, demonstrating that myosin II molecular motors are key players of the myoblast contractile forces.

Taken together, these results indicate that the contractility of the actomyosin network is enhanced in elongated myoblasts through the establishment of a dense network of parallel actomyosin fibers.

### The contractility of cell pairs increased after their fusion and raise on elongated micropatterns

**Figure 4:**
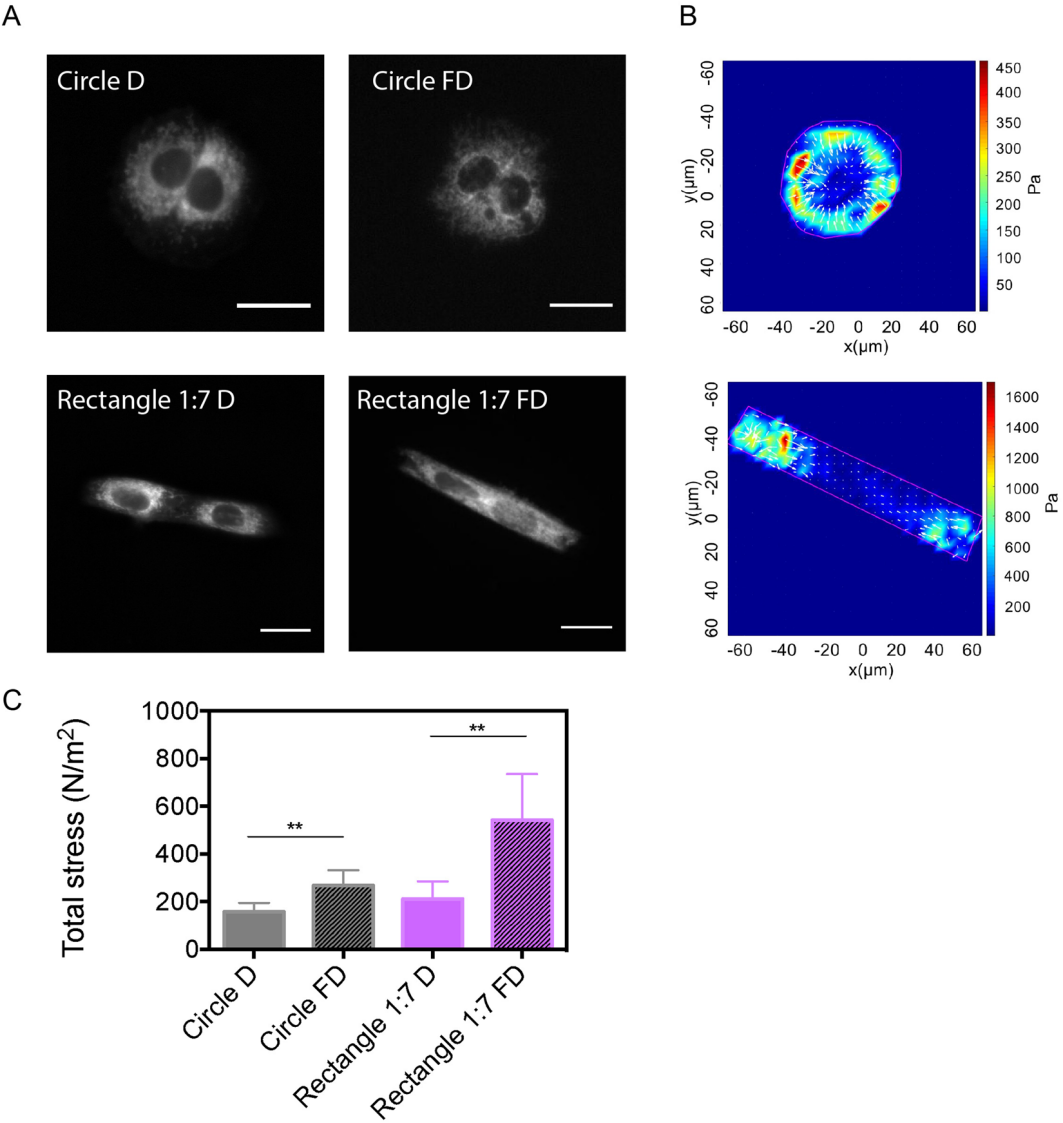
The contractility of cell pairs increased after their fusion and raise on elongated micropatterns. (**A**) Epifluorescence images of C2C12 cell pairs unfused (D) and fused (FD) on circular and rectangular (1:7 aspect ratio) FN micropatterns and stained for mitochondria with Mito Tracker. Scale bars are 20 µm. (**B**) Representative maps of the contractile stress exerted by C2C12 cell pairs fused on circular and rectangular FN micropatterns. (**C**) Evolution of the total stress for unfused (**D**), (plain bars) and fused (FD, hashed bars) C2C12 cell pairs on circular (in grey) and rectangular (in purple) FN micropatterns. Circle D: n = 8; Circle FD: n = 5; rectangle D: n = 6 and rectangle FD: n = 8. **p < 0.01.

To understand how myoblast morphology can affect contractile properties of myotubes, we consider a minimal model of two myoblasts. We first determined the traction forces exerted by cell doublets at P1 (24 h in proliferation media) plated on micropatterns of varying shapes (Supplementary Fig. 6A). There were no statistical differences of contractile stress between the wide range of CSI (Supplementary Fig. 6B), despite a well-defined orientation of the actin network (Supplementary Fig. 6C) and the nuclei (Supplementary Fig. 6D,E) on 1:4 and 1:7 rectangular micropatterns. Based on this observation, we then quantified the contractile stress exerted by cell doublets cultured on circular (CSI = 1) and rectangular (CSI = 0.50, 1:7 aspect ratio) FN micropatterns during five additional days (D5, Fig. 1H) in differentiation medium. We used MitoTracker Red CMXRos (ThermoFisher Scientific), a live mitochondrial staining, to ensure that myoblast doublets were fused (Fig. 4A)^38^. As shown in Fig. 4B,C, we found that the total contractile stress was slightly increased in fused cell doublets grown on circular micropatterns (267 ± 64 Pa) comparing to circular unfused cell doublets (157 ± 37 Pa), whereas fused elongated doublets were more than two times more contractile (543 ± 193 Pa) than unfused elongated doublets (211 ± 73 Pa). These results indicate that the cellular contractility increased after cell fusion and was significantly enhanced for elongated morphologies.

### Actomyosin contractility promotes myotube differentiation

**Figure 5:**
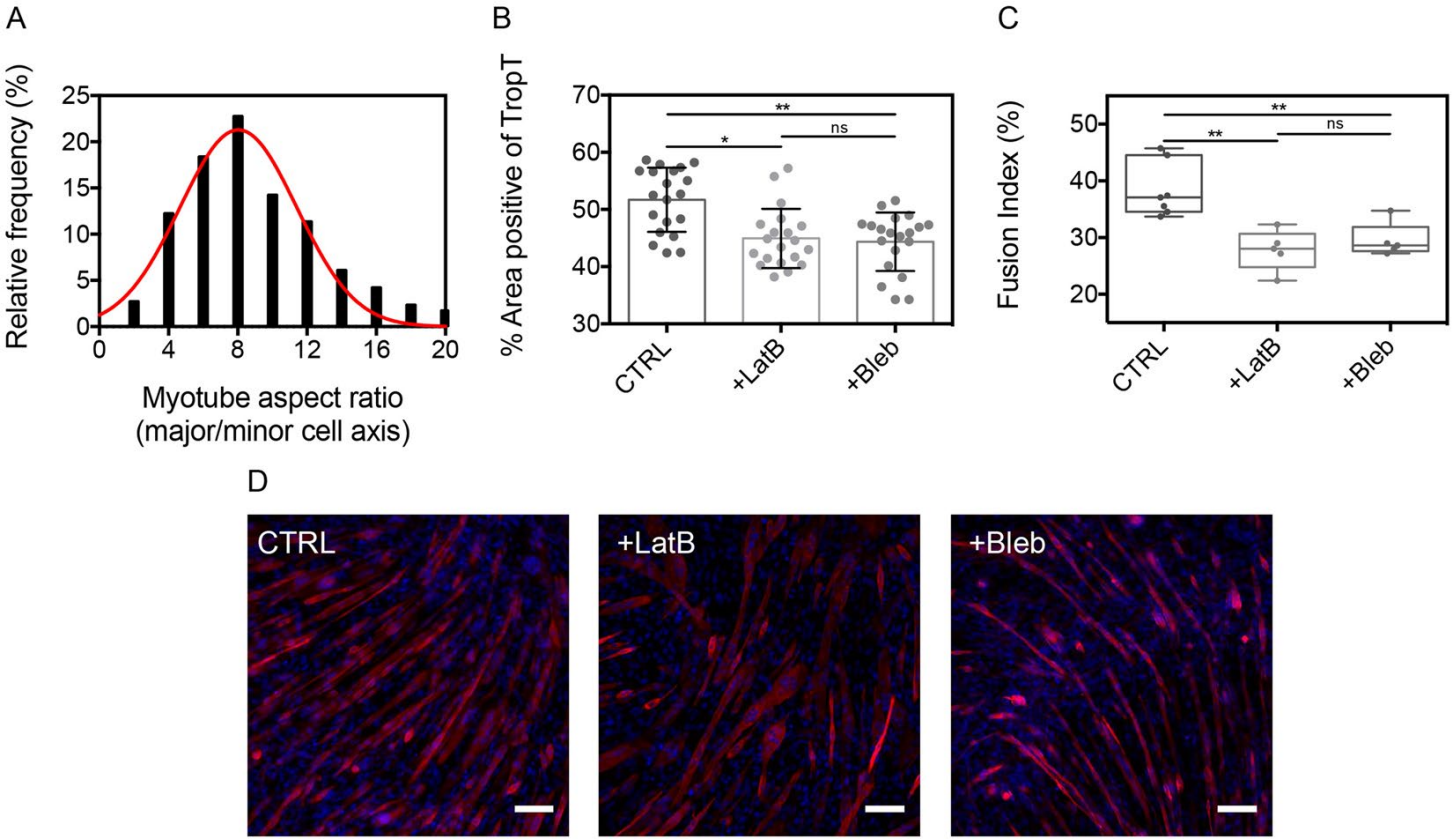
Actomyosin contractility promotes myotube differentiation. (**A**) Distribution of the myotube aspect ratio after 4 days in differentiation media (n = 114, R^2^ = 0.915). Evolution of (**B**) the percentage of positive area for troponin T and (**C**) the fusion index in control cells (CTRL), LatB and Bleb-treated cells (n = 20 for each). (**D**) Typical epifluorescence images of control myotubes (Ctrl), LatB and Bleb-treated myotubes formed after 4 days in differentiation media. Myotubes were immunostained for DNA (blue) and troponin T (red). Scale bars are 100 µm. *p < 0.05, **p < 0.01 and n.s. not significant.

We then asked whether the actomyosin contractility of myoblasts could influence myotube differentiation. C2C12 myoblasts were cultured on FN-coated substrates in proliferation media for 2 days (P2, Fig. 1H) to reach the cellular confluence. Then, either LatB or Bleb were added for 30 minutes and the proliferation medium was replaced by a differentiation culture medium. After 4 days in differentiation medium (D4, Fig. 1H), C2C12 cells were fixed and stained for DNA and TroponinT, a marker of differentiation (Fig. 5). We assessed the effect of LatB and Bleb treatments by staining alpha actinin on control myotubes (CTRL) and myotubes treated with Latrunculin B (+LatB) and blebbistatin (+Bleb). As observed in Supplementary Fig. 7, control myotubes present typical striated Z-band patterns, whereas LatB and Bleb treatments disturb striation patterns in myotubes. Control myotubes (CTRL) were characterized by a mean aspect ratio of 7.99 ± 3.38 (Fig. 5A) and a positive Troponin T area of 51.7 ± 5.6% (Fig. 5B). Our findings indicate that LatB and Bleb treatments decreased the fraction of Troponin T area to 44.9 ± 5.2% and 44.4 ± 5.1%, respectively. In addition, we found that the fusion index was statistically lower for LatB (27 ± 3%) and Blebb-treated (29 ± 3%) cells than for control cells (38 ± 5%), strengthening the role of the actomyosin contractility in myoblast differentiation (Fig. 5C). Taken together, these results indicated that the actomyosin contractility of myoblasts promotes myotube differentiation.

### Myoblast elongation triggers YAP nuclear export, which is essential to the differentiation of myoblasts into myotubes

**Figure 6:**
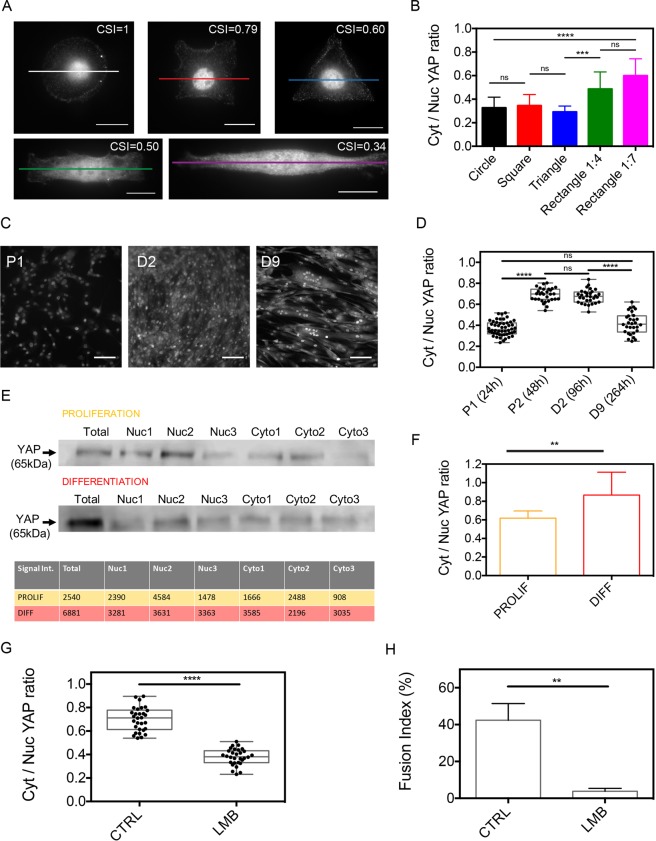
Myoblast elongation triggers YAP nuclear export, which is essential to the differentiation of myoblasts into myotubes. (**A**) Epifluorescence images of C2C12 cells grown on FN micropatterns of 1600 µm^2^ and immunostained for YAP. Scale bars are 20 µm. (**B**) Cytoplasmic to nuclear YAP ratio for the different myoblast morphologies (n = 10 for each). (**C**) Epifluorescence images of confluent C2C12 cells immunostained for YAP at day 1 (P1, 24 h) of the proliferation stage and at day 2 (D2, 96 h) and day 9 (D9, 264 h) of the differentiation stage. Scale bars are 100 µm. (**D**) Cytoplasmic to nuclear YAP ratio at P1 (n = 50), P2 (n = 30), D2 (n = 30) and D9 (n = 30). (**E**) Western blot analysis of YAP (65 kDa) expression in C2C12 proliferation and differentiation. (**F**) Cytoplasmic to nuclear YAP ration (mean ± S.D.) during proliferation and differentiation (3 replicates for each condition with 20.10^6^ cells/replicate). (**G**) Cytoplasmic to nuclear YAP ratio at D2 and (**H**) fusion index for control (CTRL) and leptomycin (LMB)-treated cells at D4. **p < 0.01, ****p < 0.0001 and n.s not significant.

To gain more insight into the intracellular mechanisms that control the myotube differentiation, we consider the role of YAP by determining its localization in either the cytoplasm or the nucleus of myoblasts, where it binds to and activates TEAD transcription factors^39^. To this end, we quantified the cytoplasmic/nuclear YAP ratio in micropatterned myoblasts (Fig. 6A and Supplementary Fig. 8). Cell elongation on 1:4 and 1:7 rectangular micropatterns increased the cytosolic/nuclear YAP ratio (Fig. 6B), suggesting that cell elongation triggers YAP nuclear export through nuclear compressive forces exerted by the actomyosin network^40^. To verify whether the localization of YAP in either the cytoplasm or the nucleus is related to specific stages of the differentiation process, we stained YAP in C2C12 cells after 24 h (P1) and 48 h (P2) of the proliferation step and at 96 h (D2) and 264 h (D9) of the differentiation step (Fig. 6C and Supplementary Fig. 9). Interestingly, our results indicated that the cytoplasmic/nuclear YAP ratio increased from 0.37 ± 0.07 at P1 to 0.68 ± 0.06 at P2 and 0.67 ± 0.06 at D2 and then decreased to 0.42 ± 0.10 at D9 (Fig. 6D). Interestingly, the cytoplasmic/nuclear YAP ratio at D9 was found to be statistically not different from P1 values, suggesting that the cytoplasmic/nuclear YAP ratio in differentiated myotubes is similar to that of myoblasts. To verify these results, we harvested myoblasts at the proliferation stage P1 (24 h) and the differentiation stage D2 (96 h) and we isolated their cytoplasmic and nuclear materials. We carried out a bicinchoninic acid (BCA) protein assay and the proteins contained in cytoplasmic and nuclear extractions were analyzed by electrophoresis (Fig. 6E). Our Western blot findings indicated that the cytoplasmic/nuclear YAP ratio increased from 0.62 ± 0.08 at P1 to 0.87 ± 0.24 at D2 (Fig. 6F). This biochemical quantification of YAP demonstrated a significant YAP nuclear export in differentiating C2C12 cells and strengthen our findings.

Based on these results, we assessed the role of YAP by using leptomycin B to block active export from the nucleus by directly binding to exportin1^41^. Indeed, Alberto Elosegui-Artola and coworkers have shown recently that leptomycin B (LMB) can be used efficiently to study how contractile forces exerted by the cytoskeleton drive YAP nuclear translocation^39^. By treating C2C12 myoblasts at P2 (48 h) with LMB, we found that the cytoplasmic to nuclear YAP ratio was significantly lower in LMB-treated cells at D2 (96 h, Fig. 6G), suggesting that YAP is mainly concentrated within the nucleus when the nuclear export is inhibited. In addition, our findings indicated that the fusion index at D4 of LMB-treated cells (Fig. 6H) was very low (3.8 ± 1.5%) compared to control cells (42.4 ± 9.1%), demonstrating that active nuclear export is required for C2C12 differentiation.

Taken together, our findings show that YAP nuclear export in muscle cells is a reversible process and corresponds to the fusion/differentiation stages (from P2 to D2), suggesting that YAP signaling is required for the differentiation of C2C12 myoblasts into myotubes.

## Discussion

In addition to gene expression, biochemical factors or dimensionality of the cell microenvironment, the balance of mechanical forces that regulate morphology and spatial localization of cells participates actively to shape the tissues in the human body^42^. It is now well established that mechanical forces and cell shape direct cell fate and regulate cell cycle progression^25,43–45^. The tissue of skeletal muscles is composed of bundles of multinucleated muscle fibers. The fusion of hundreds or thousands of mononucleated muscle myoblasts is required to form each myofiber^46^. Myoblast fusion is a critical physiological step, which is required not only for skeletal muscle development during embryogenesis, but also for the regeneration of injured muscle tissues in adults by satellite cells^47^. During the fusion process, the cell membranes of two neighbouring myoblasts are brought into close proximity by the interplay between protrusive and resisting forces exerted by both fusing myoblasts. During the last decade, a biophysical framework has emerged suggesting that protrusive and resisting forces put fusogenic synapses under high mechanical tension to drive cell membrane fusion^48^. Despite these findings, the specific role of cell shape, actomyosin contractility and Hippo pathway member YAP in myoblast differentiation was yet to be established.

*In vivo* mesenchymal cells provide a population of mononucleated myoblasts known to be the precursors of contractile muscle cells. Myoblasts progressively fuse to form plurinucleate syncytia named myotubes^48^, which furtherly differentiate to acquire the final morpho-functional features of muscle cells^49^. We showed that C2C12 myoblasts, which are characterized by a wide range of morphologies *in vitro*, progressively change their shape during the fusion/differentiation process to adopt an elongated and fusiform morphology. Interestingly, we found that the amount of F-actin in C2C12 myoblasts was proportional to their spreading area. Taken together, our results demonstrate that both cell shape and spreading area control the mechanical state of myoblasts.

By using a microcontact printing technique to standardize the morphology of single myoblasts^50^, we demonstrated that the spatial organization of both the actin network and the nucleus was directed by the myoblast morphology. We found that elongated myoblasts were characterized by a well-organized actin cytoskeleton that forms thick bundles of actin fibers oriented parallel to the long cell axis. As observed previously on endothelial cells^40^, actomyosin fibers exert compressive forces on both sides of the nucleus of myoblasts, leading to large nuclear deformations and the orientation of the nucleus with respect to the long cell axis. These findings suggest that the fusiform morphology of myoblasts is accompanied by a significant remodeling of both the actomyosin network and the nucleus. This mechanical model is supported by the quantification of the traction stress exerted by myoblasts of varying morphologies, demonstrating that actomyosin contractility in myoblasts is enhanced by the cellular elongation rate. Myoblast elongation induces a significant increase of tension in actomyosin cables that regulate the nuclear shape through lateral compressive forces^40^, suggesting a mechanistic regulation between cell and nuclear shape in myoblasts. To demonstrate that myoblast elongation is critical to the differentiation step, we then determined the contractile stress of cellular doublets formed on circular and rectangular micropatterns. Using this minimal model of myotubes, our findings demonstrate that the cellular contractility was significantly enhanced after the fusion of elongated cell pairs, suggesting that myoblasts must adopt a fusiform shape to fuse and acquire the highest level of cell contractility. By using LatB and Bleb drugs, we showed that high internal stresses produced by long actomyosin cables in elongated myoblasts promote the differentiation of myoblasts into myotubes.

An important question that was needed to be addressed was whether large nuclear deformations observed in elongated myoblasts may affect mechanotransduction pathways. To address this question, we quantified YAP, which is a mechanosensitive transcriptional regulator^39,51^ that was recently found to play a key role in mechanotransduction in skeletal muscles by modulating myogenesis and muscle regeneration^52^. In addition, YAP activity has been also reported in muscular dystrophy. YAP expression increases during satellite cell activation and YAP must be phosphorylated for differentiation of C2C12 myoblasts into myotubes^30^. Consistent with the role of YAP in muscle regeneration, our findings show that YAP nuclear export is enhanced during both fusion and differentiation stages, suggesting that the cytoplasmic expression of the YAP Hippo pathway member is required to promote the differentiation of myoblasts into myotubes. After differentiation into myotubes, YAP is mostly expressed in the nucleus, as observed in myoblasts that start to proliferate. We found that the myoblast elongation triggers YAP nuclear export, increasing evidence that YAP is a key regulator of mechanotransduction in myoblasts. Collectively, our findings support a mechanical model where actomyosin contractility scales with myoblast elongation and enhances differentiation in myotubes through YAP nuclear export.

## Methods

### Cell culture

C2C12 myoblasts (American Type Culture Collection), immortalized by D. Yaffe and O. Saxel in 1977^53^, were maintained in polystyrene flasks in a 37 °C, 5% CO2 incubator, and cultured in proliferation medium composed of DMEM (Dubelcco’s Modified Eagle’s medium) High Glucose (4.5 g/l) with L-glutamine (BE12–604F, Lonza) supplemented with 10% FBS (Fetal Bovine Serum, AE Scientific) and 1% antibiotic (Penicillin/Streptomycin, AE Scientific). C2C12 cells were differentiated in differentiation medium containing DMEM High Glucose with L-glutamine, 2% HS (Horse Serum, VWR) and 1% antibiotic. Cells were seeded on micropatterned substrates at a density of 20,000 cells per well. The proliferation medium was replaced with differentiation medium after 24 hours and then half of the medium was replaced daily. Immortalized human myoblasts 16Ubic (Paul D. Wellstone Muscular Dystrophy Cooperative Research Center for FSHD, University of Massachusetts Medical School, UMMS) come from the biceps (bic) of a patient (woman of 60 years old, numbered 16) unaffected (U) by the Facioscapulohumeral muscular dystrophy, FSHD, and who have been confirmed to lack a 4qA deletion. Human 16Ubic myoblasts were cultured in proliferation medium composed of DMEM High Glucose (4.5 g/l) with L-glutamine supplemented with 20% medium 199-glutaMAX (ThermoFisher Scientific, Watltham, MA USA 02451), 10% FBS (AE Scientific), 1% Ultroser (PALL, New-York, NY USA 11050), 2% 1 M HEPES (ThemoFisher Scientific), 0.05% zinc sulfate (Merck, Germany), 0.01% Vitamin B12 (Merck, Germany) and 1% antibiotic^54,55^.

### Cell culture substrates and microcontact printing

A polydimethylsiloxane (PDMS, Sylgard 184 from Dow Corning) mixture of 10:1 monomer:crosslinker ratio was spin-coated on glass coverslips previously washed in a 70:30 ethanol:water solution^56^. PDMS was then cured at 60 °C for 4 hours to obtain a thin membrane of 3 MPa and ~60 µm thick^57^. Hydroxy-polyacrylamide (hydroxy-PAAm) hydrogels of 12kPa in Young’s modulus were fabricated by mixing acrylamide (AAm, A8887, Sigma), bisacrylamide (bis-AAm, 146072, Sigma) and N-hydroxyethylacrylamide (HEA, 697931, Sigma) monomers^19,20,58^. The polymerization was initiated with ammonium persulfate (APS) and N, N, N′, N′-Tetramethylethylenediamine (TEMED). Both PDMS and hydroxy-PAAm hydrogels were functionalized with protein islands by microcontact printing^59^. PDMS stamps were prepared by casting microstructured silicon wafers with a 10:1 monomer:crosslinker ratio. PDMS was then cured at 60 °C for 4 hours. The thick PDMS membrane was then peeled off and the stamps were carefully cut and washed in an ultrasonic bath (Bandelin sonorex digitec) for 15 minutes using in a 5% solution of detergent (Decon 90, Decon Laboratories Limited). PDMS stamps were rinsed three times with demineralized water and finally with a 70% ethanol solution. PDMS stamps were made hydrophilic under ultraviolet-ozone (UV/O3) expositions for 7 minutes. Then a solution of fibronectin (FN, Millipore Corp.) and poly-l-lysine (PLL, Sigma) was incubated under a sterile hood on the structured side of the stamps for 1 hour^60^. PDMS stamps were gently dried with a nitrogen flow and placed into contact with PDMS coated coverslips or hydroxy-PAAm hydrogels for 30 seconds or 1 hour respectively to obtain adhesive micropatterns of 1600 µm^2^ that span a wide range of geometries: circle, square, triangle and rectangle of 1:4 and 1:7 aspect ratios. Microprinted PDMS and hydroxy-PAAm substrates were then passivated with a pluronic F-127 solution (BASF) and a BSA solution (GE Healthcare), respectively.

### Immunostaining

C2C12 myoblasts were fixed and permeabilized with 4% paraformaldehyde (Electron Microscopy Sciences), 0,05% Triton X-100 (Sigma) in Phosphate buffered saline (PBS 1X, Capricorn scientific) for 12 min at room temperature. Fixed cells were rinsed three times in warm PBS and incubated 30 min with a blocking solution containing 1% BSA (Bovine Serum Albumine, GE Healthcare) and 5% FBS in PBS. C2C12 myoblasts were labeled for F-actin (Alexa Fluor 488 Phalloidin, 1:200, Molecular Probes), DNA (DAPI, 1:200, ThermoFisher Scientific D1306), and vinculin (Monoclonal anti-vinculin antibody produced in mouse, 1:200, HVIN-1 clone, Sigma).

Yap was labeled with YAP1 monoclonal antibody (produced in mouse (M01), clone 2F12, Abnova), TroponinT with Anti troponin-T produced in mouse (1:200, Sigma-Aldrich) and alpha-actinin with monoclonal anti alpha actinin produced in mouse (1:200, Sigma-Aldrich) for 45 min at 37 °C. Myoblasts were washed three times in PBS, incubated with an anti-mouse antibody produced in goat and labeled with a goat anti-mouse antibody (1:200, Molecular Probes, tetramethylrhodamine, Invitrogen, T2762) for 45 min at 37 °C. Immunostained cells were mounted on microscope slides with SlowFade Gold Antifade (Thermofisher, Molecular probes) for epifluorescence and confocal imaging. Mitochondria was labeled in living cells with MitoTracker Red CMXRos (ThermoFisher Scientific) for 30 minutes at 50 nM in proliferation media. After incubation, the medium was replaced with fresh medium.

### Immunodetection by Western Blot

At first, we harvested proliferating myoblasts (P1–24h) and at the beginning of differentiation (D2–96h). For this, the cells were scrapped on ice and harvested in 1 ml PBS. Then, the cytoplasmic and nuclear extraction was performed using the NE-PER Nuclear and cytoplasmic extraction reagents kit (ThermoScientific). After isolating cytoplasm and nuclei from proliferating cells and differentiating cells, a BCA protein assay (Pierce BCA Protein Assay Kit, ThermoScientific) was carried out. The different proteins contained in the cytoplasmic and nuclear extractions were then migrated by electrophoresis on polyacrylamide gel and transferred on nitrocellulose. The immunodetection of the YAP protein was carried out by the addition of a primary antibody (YAP1 monoclonal antibody produced in mouse (M01), clone 2F12, Abnova) followed by the recognition by a secondary antibody coupled to horseradish peroxidase (ECL Anti-mouse IgG HRP, GE Healthcare).

### Epifluorescence and confocal imaging

C2C12 myoblasts were observed in epifluorescence and confocal mode with a Nikon Eclipse Ti-E motorized inverted microscope equipped with ×60 and ×100 Plan Apo (NA 1.45, oil immersion) objectives, two lasers (Ar ion 488 nm; HeNe, 543 nm) and a modulable diode (408 nm) and recorded with a Roper QuantEM:512SC EMCCD camera (Photometrics Tucson, AZ) using NIS Elements Advances Research 4.0 software (Nikon). Morphometric analysis of focal adhesions (area, perimeter and shape factor) was conducted using a custom-made Matlab code and confocal images using small Z-depth increments between focal sections (0.15 μm) were processed using NIS-Elements (Nikon, Advanced Research version 4.0).

### Traction force microscopy

To determine the tensile stresses exerted by myoblasts of different shapes, C2C12 cells were cultured on micropatterned hydroxy-PAAm hydrogels containing fluorescent microbeads of 0.2 µm in diameter (Fluospheres, Molecular Probes, Eugene, OR)^33^. Cells were detached with a trypsin treatment (1:10 in PBS, Capricorn Group) and the bead displacements in the hydrogel were recorded during the cell detachment. The displacement field was determined by comparing the position of the beads before (when the cell exerts forces) and after (when the substrate is relaxed) the cell detachment. The bead displacements were measured by particle image velocimetry (PIV). A grid consisting of squares of 16 × 16 pixels were superimposed on the pictures (512 × 512 pixels) of the beads before and after detachment. The general movement of the beads inside each square was associated to a vector placed at the center of each square. We considered only horizontal bead displacements (xy plan), which were assumed to be smaller than the thickness of the substrate that was considered as a semi-infinite medium. The field of displacements can be described as the convolution between the point forces and the Green tensor:

$${d}_{i}=\mathop{\sum }\limits_{j=1}^{m}\,G({r}_{ij}){F}_{j}$$

where di is the displacement at point xi, Fj is the force at each point xj, and G(rij) correspond to the Green tensor (with rij = xi − xj). The resolution of this equation being complex, the Fourier Transform (FT) will be applied and the displacements in the Fourier space can then be written:

$${d}^{\ast }(k)={G}^{\ast }(k)\cdot {F}^{\ast }(k)$$

By applying a FT, the convolution becomes a simple multiplication where k corresponds to the radial wave vector and the * represent the Fourier space. The expression of the Green tensor in the Fourier space is:

$${G}^{\ast }(k)=\frac{2(1+\nu )}{E{k}^{3}}(\begin{array}{cc}(1-\nu ){k}^{2}+\nu {k}_{y}^{2} & \nu {k}_{x}{k}_{y}\\ \nu {k}_{x}{k}_{y} & (1-\nu ){k}^{2}+\nu {k}_{x}^{2}\end{array})$$

where k = (kx, ky), E is the Young modulus (E = 12 kPa) and *ν* is the Poisson coefficient (~0.5).

In this Fourier space, the traction forces can be expressed as follow:

$${F}^{\ast }(k)={d}^{\ast }(k)\cdot {G}^{\ast }{(k)}^{-1}$$

Finally, an inversed Fourier transform was performed to determine the traction force maps. All the forces not included in the cell boundary were set to zero. The field of traction forces was corrected, and the measured displacements were replaced by the displacements obtained from the corrected traction forces. Successive iterations were performed until the traction forces within the cell converge.

### Pharmacological treatments

C2C12 cells were incubated with latrunculin B (Lat B, Abcam) for 30 min at 0.5 µM in DMSO to disrupt the actin network and Blebbistatin (Bleb, Enzo Life Sciences) at 25 µM in DMSO was used to inhibit non-muscle myosin II molecular motors. After treatment, myoblasts were fixed and stained. Control experiments in DMSO were performed to verify that DMSO does not affect the cytoskeleton. Leptomycin B, inhibitor of nuclear export was diluted at 20 nM in the proliferation medium and was added to C2C12 myoblasts after 48 h in proliferation (P2). Because of the reversible effect of the leptomycin B, the drug was also diluted at 20 nM in the differentiation medium and added to the C2C12 cells in differentiation.

### Statistical analysis

Differences in means between groups were evaluated by two-tailed Student’s t-tests performed in Prism 7b (Graphpad Software, Inc.). For multiple comparisons the differences were determined by using an analysis of variance (ANOVA) followed by Tukey post-hoc test. *p < 0.05, **p < 0.01, ***p < 0.001, ****p < 0.0001 and n.s. not significant. Unless otherwise stated, all data are presented as mean ± standard deviation (S.D.).

## Supplementary information

Supplementary Informations
