# Supplementary material for: Actomyosin contractility scales with myoblast elongation and enhances differentiation through YAP nuclear export
Source: Sci Rep. 2019 Oct 29;9:15565. doi: 10.1038/s41598-019-52129-1 (PMC6820726; doi:10.1038/s41598-019-52129-1)
Supplement: Supplementary file 1 — Supplementary Informations [file 41598_2019_52129_MOESM1_ESM.pdf]

Supplementary information

Actomyosin contractility scales with myoblast elongation and enhances their differentiation through YAP nuclear export

Céline Bruyère[a], Marie Versaevel[a], Danahe Mohammed[a], Laura Alaimo[a], Marine Luciano[a], Eléonore Vercruysse[a] and Sylvain Gabriele[a,1]

[a] University of Mons, Laboratory for Complex Fluids and Interfaces, Mechanobiology and Soft Matter group, Research Institute for Biosciences, Place du Parc, 20 B-7000 Mons, Belgium

[1]To whom correspondence should be addressed. Email: sylvain.gabriele@umons.ac.be

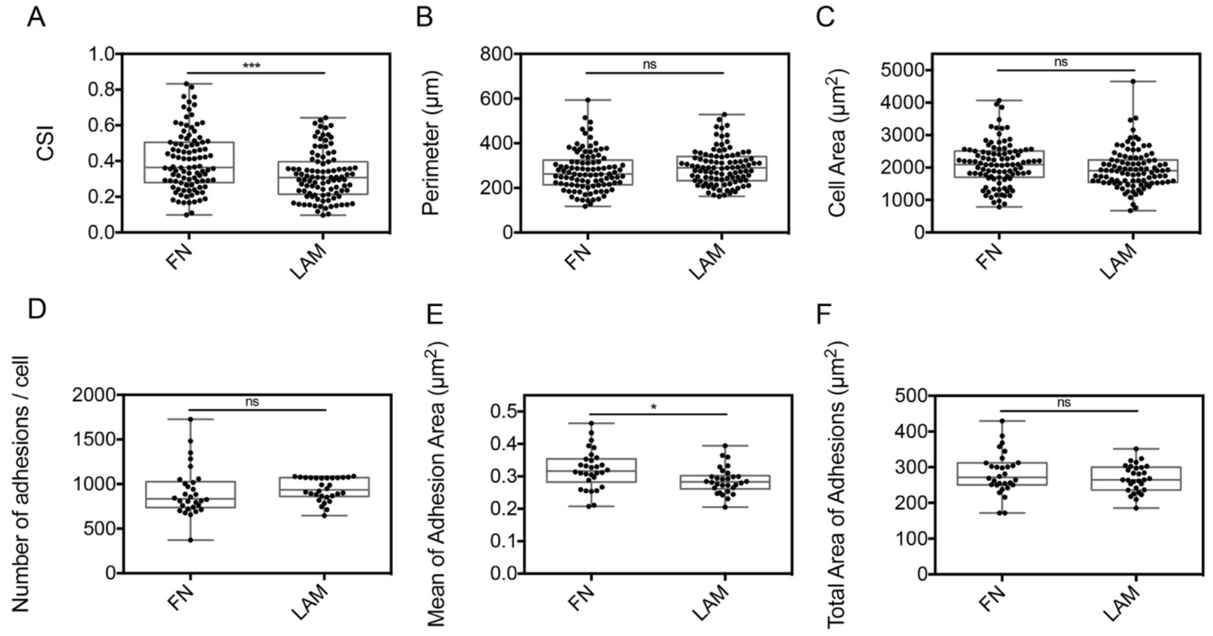

Figure S1 – Behaviour of C2C12 myoblasts on FN and LM coatings: (A) cell shape index, (B) cell perimeter, (C) cell area, (D) number of adhesions per cell, (E) mean of adhesion area and (F) total area of adhesions.

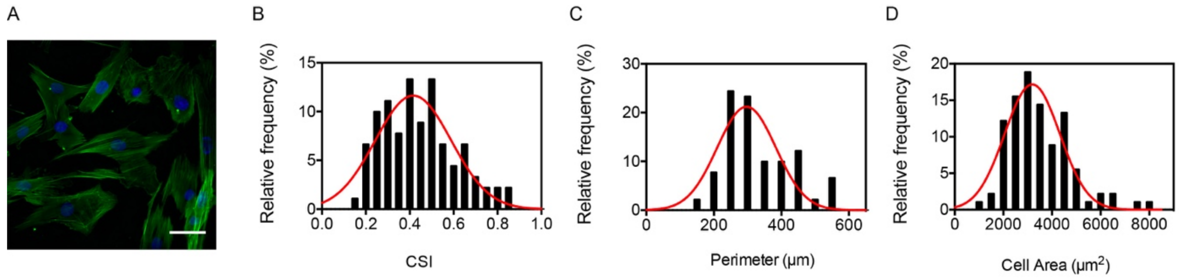

Figure S2 – (A) Epifluorescence image of human myoblasts (16 UBic) immunostained after 24 hours in culture for actin filaments (in green) and DNA (in blue). The scale bar is 20 µm. Relative frequency of (B) cell shape index, (C) cell perimeter and (D) cell area (n=90 for each).

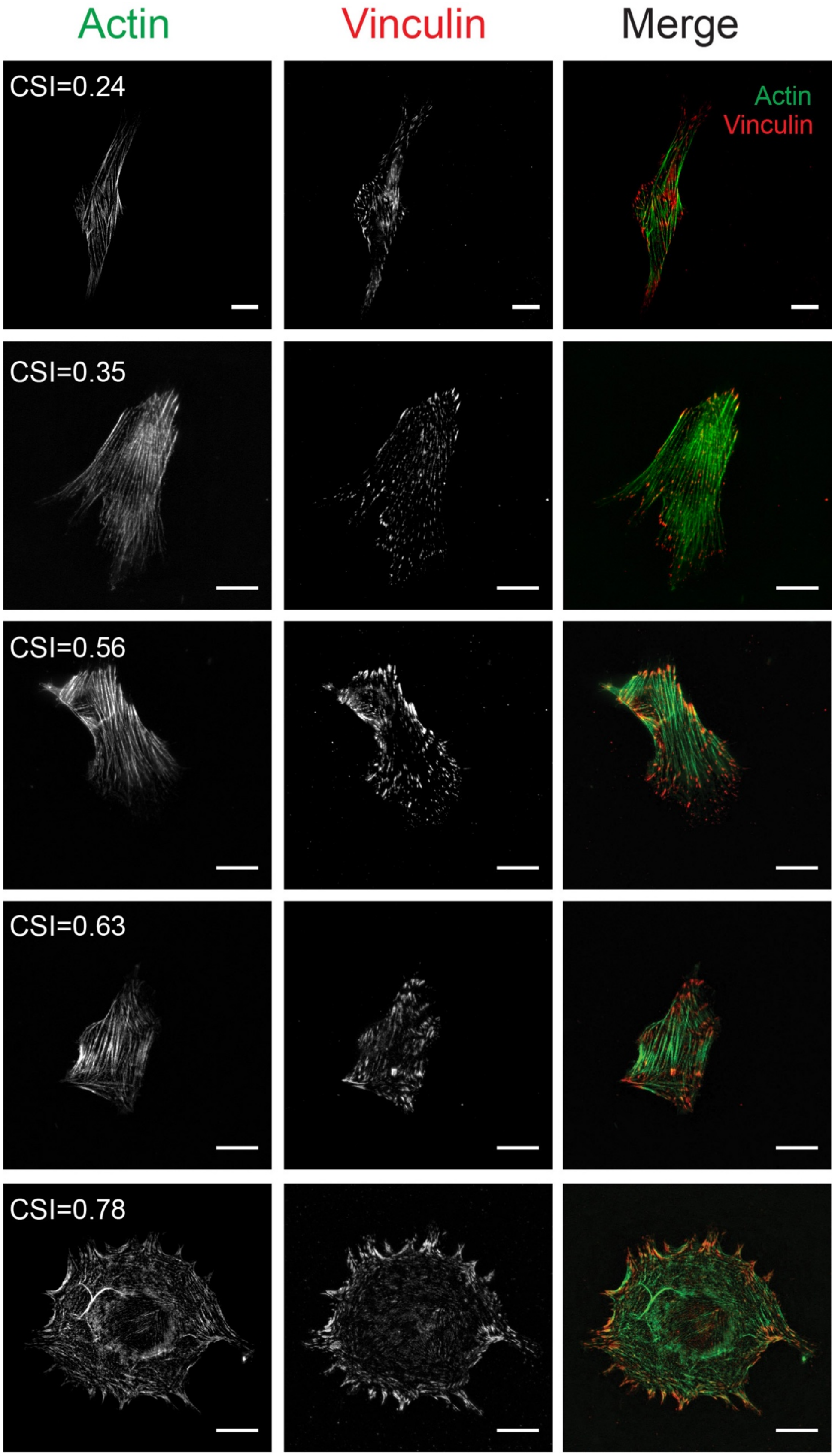

Figure S3 - Epifluorescent images of individual C2C12 myoblasts stained for actin (green) and vinculin (red). CSI values ranging from 0.24 to 0.78 characterize the different morphologies adopted by myoblasts *in vitro*. Scale bars are 20 µm.

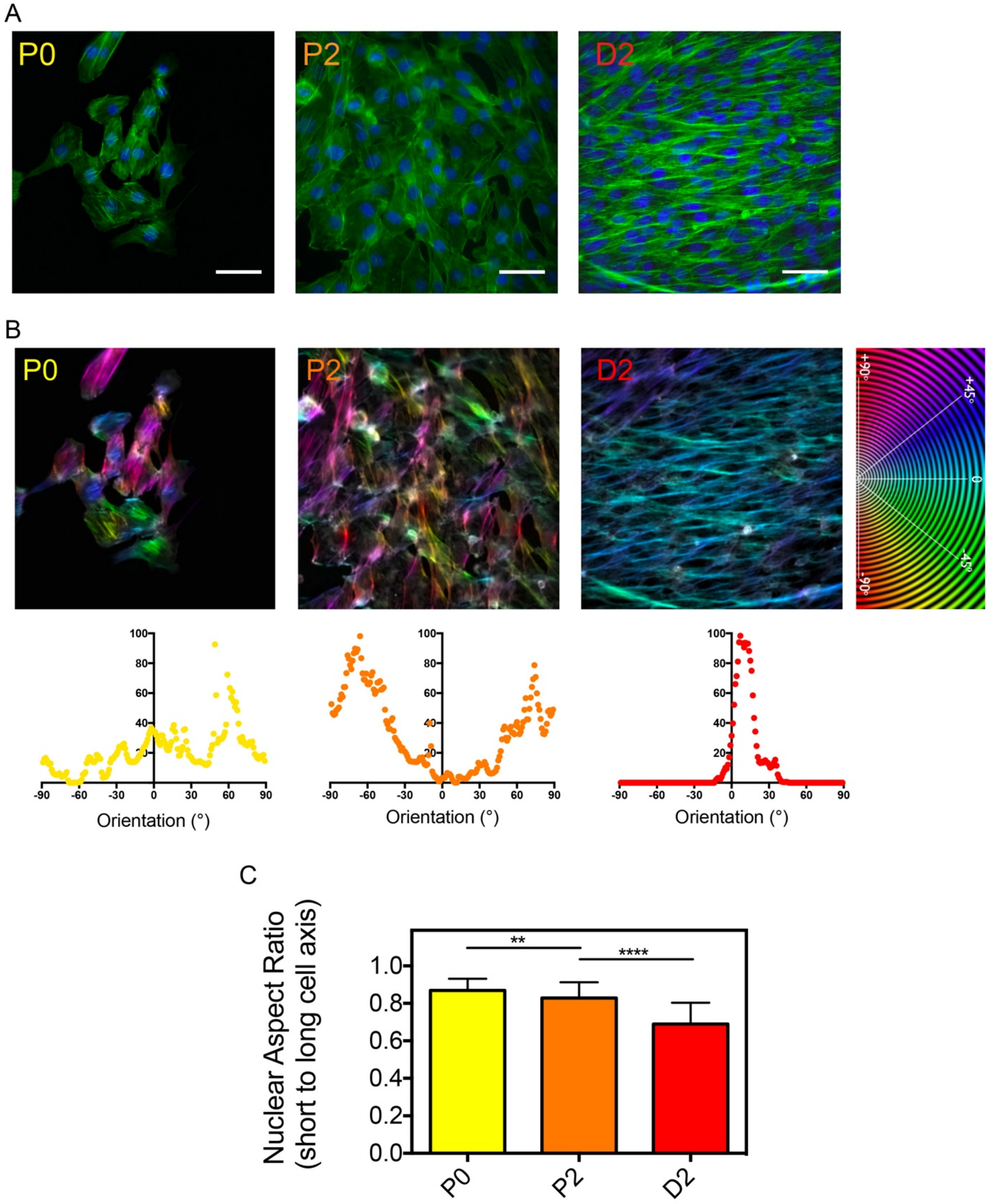

Figure S4 – (A) Epifluorescent images of myoblasts at P0 (6 hours), P2 (48 hours) and D2 (96 hours). Actin filaments are stained in green with Alexa Fluor 488 Phalloidin and DNA in blue with DAPI. Scale bars are 50 µm. (B) Orientation of actin filaments at P0, P2 and D2 is colour-coded. (C) Evolution of the nuclear aspect ratio in myoblasts at P0 (n=66), P2 (n=161) and D2 (n=70). **$p < 0.01$, ****$p < 0.0001$.

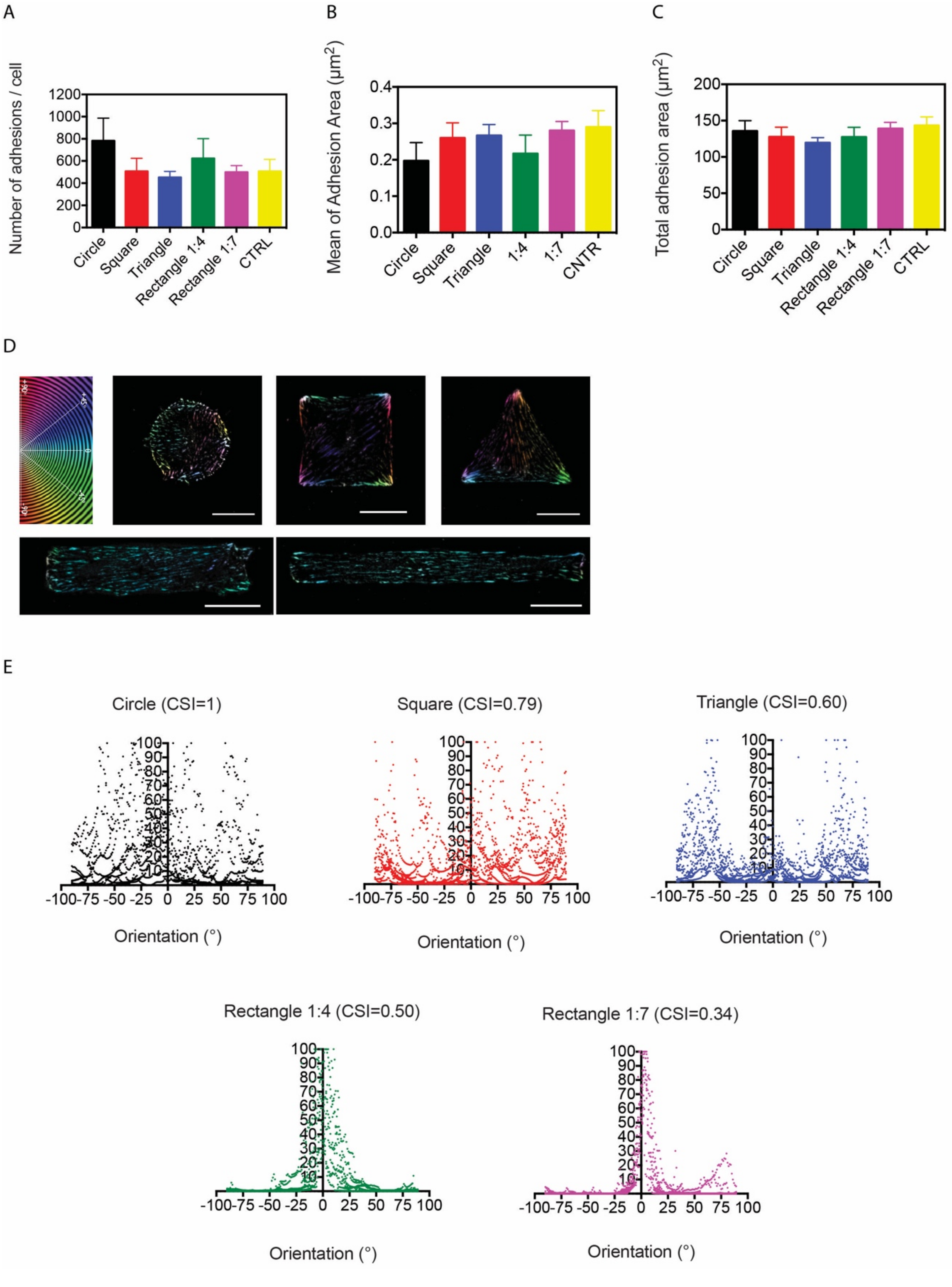

Figure S5 - Evolution of (A) the mean number of adhesions per cell, (B) the mean area per focal adhesion and (C) and the total adhesion area of adhesions for C2C12 myoblasts of various morphologies (n=10 for each). (D) Typical examples of the spatial organization of the vinculin containing cell-substrate adhesions in micropatterned C2C12 cells. Vinculin adhesions were color-coded according to their orientations. (E) Orientation of the adhesions in rounded, squared, triangular and rectangular (1:4 and 1:7 aspect ratio) micropatterned myoblasts (n=10 for each).

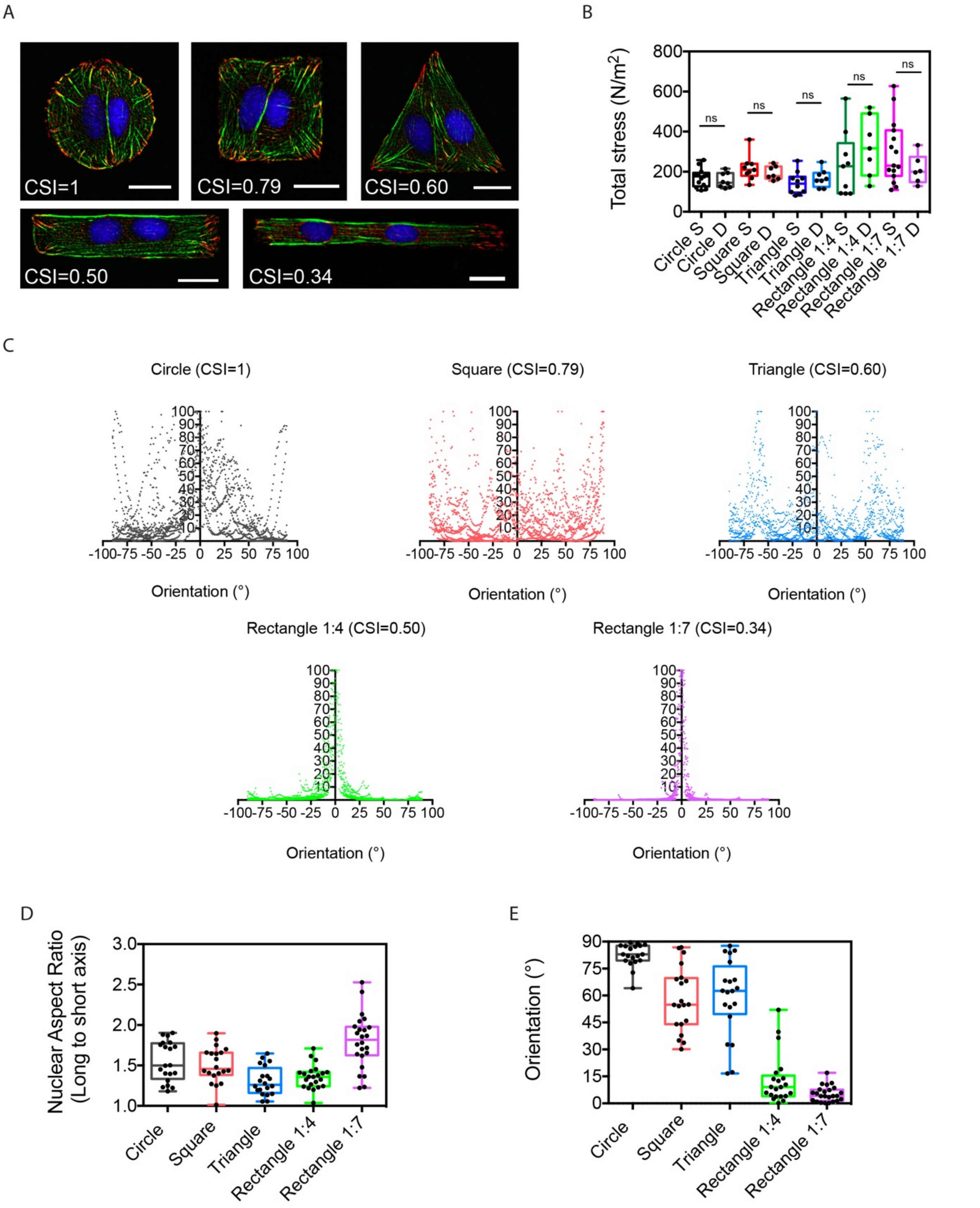

Figure S6 - (A) Epifluorescence images of C2C12 doublet cells grown on fibronectin (FN) micropatterns of 2500 µm² and immunostained for actin (green), nuclei (blue) and vinculin (red). Scale bars are 20 µm. (B) Evolution of the total stress for different morphologies of single (name-S) or doublet (name-D) cells grown on FN micropatterns. (C) Orientation of the actin network in rounded (n=10 in grey), squared (n=11 in red), triangular (n=10 in blue), rectangular 1:4 (n=12 in green) and 1:7 (n=12 in purple) micropatterned myoblasts. Evolution of (D) the nuclear aspect ratio and (E) the nuclear orientation for the different micropattern geometries ($20 \leq n \leq 24$).

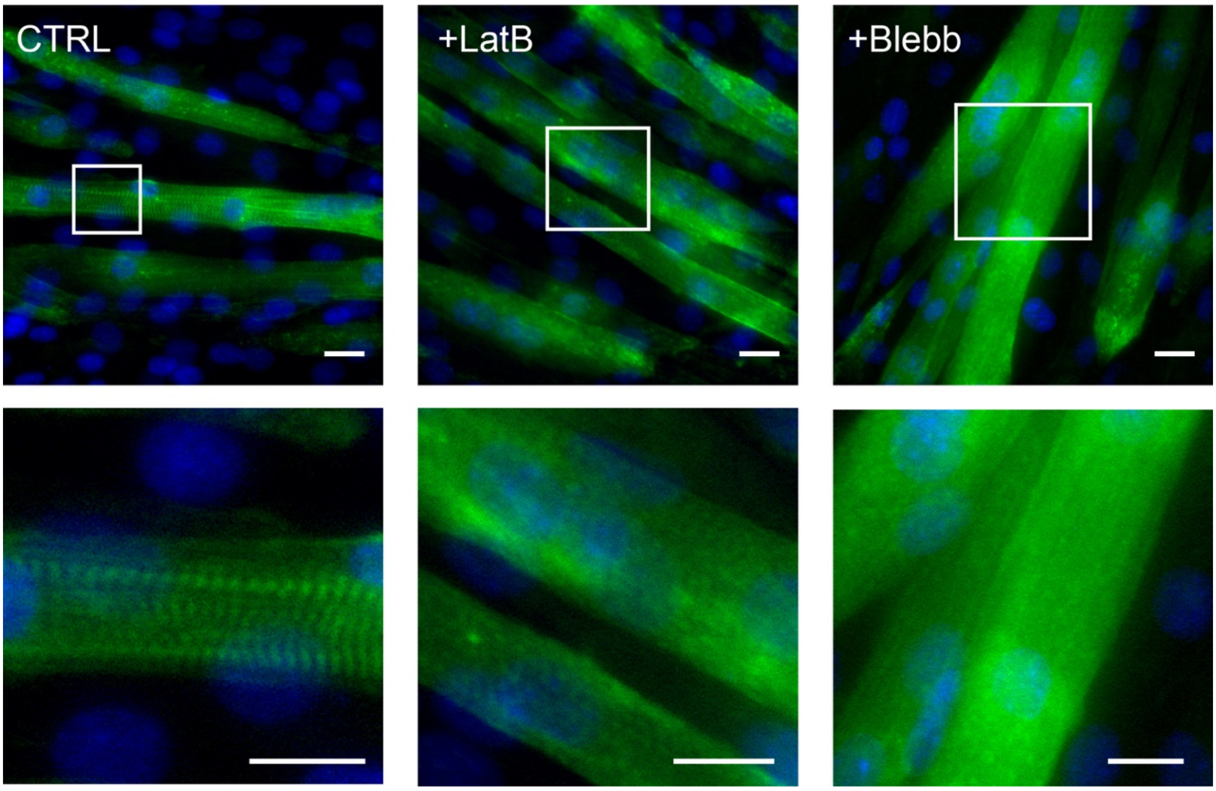

Figure S7 – Epifluorescence images of control myotubes (CTRL) and myotubes treated with Latrunculin B (+LatB) and blebbistatin (+Bleb). Alpha-actinin is labelled in green and DNA is labelled in blue. The scale bar is 20 µm.

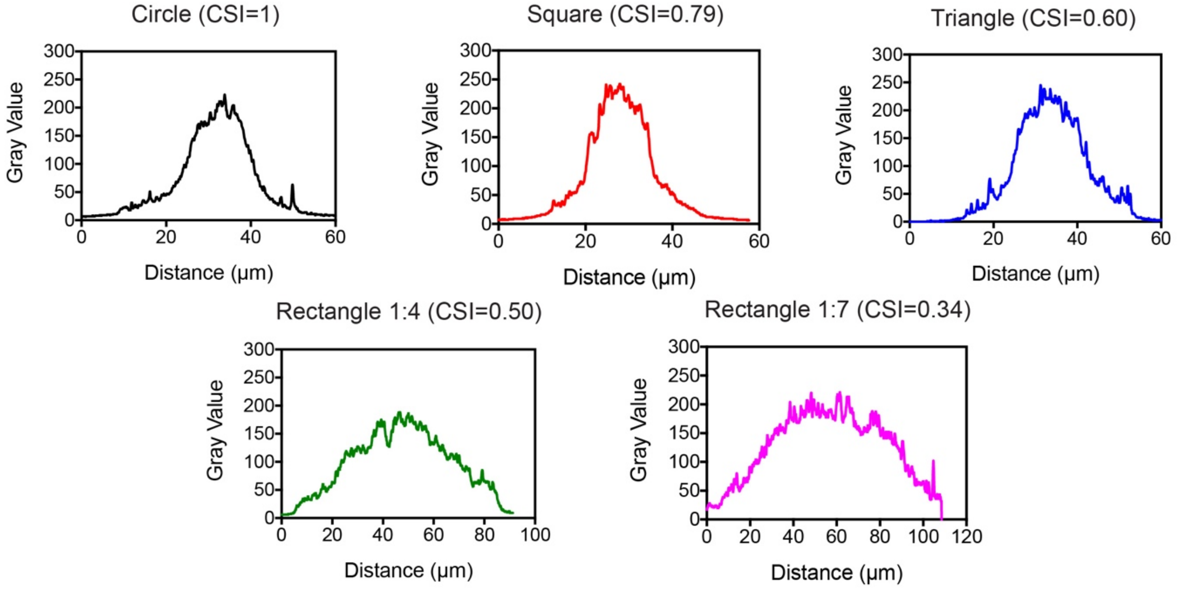

Figure S8 – Typical plot profile distribution of YAP for the different myoblast morphologies (circle in black, square in red, triangle in blue, 1:4 rectangle in green and 1:7 rectangle in purple). Coloured segments in Fig. 6A indicate the plot profile axis.

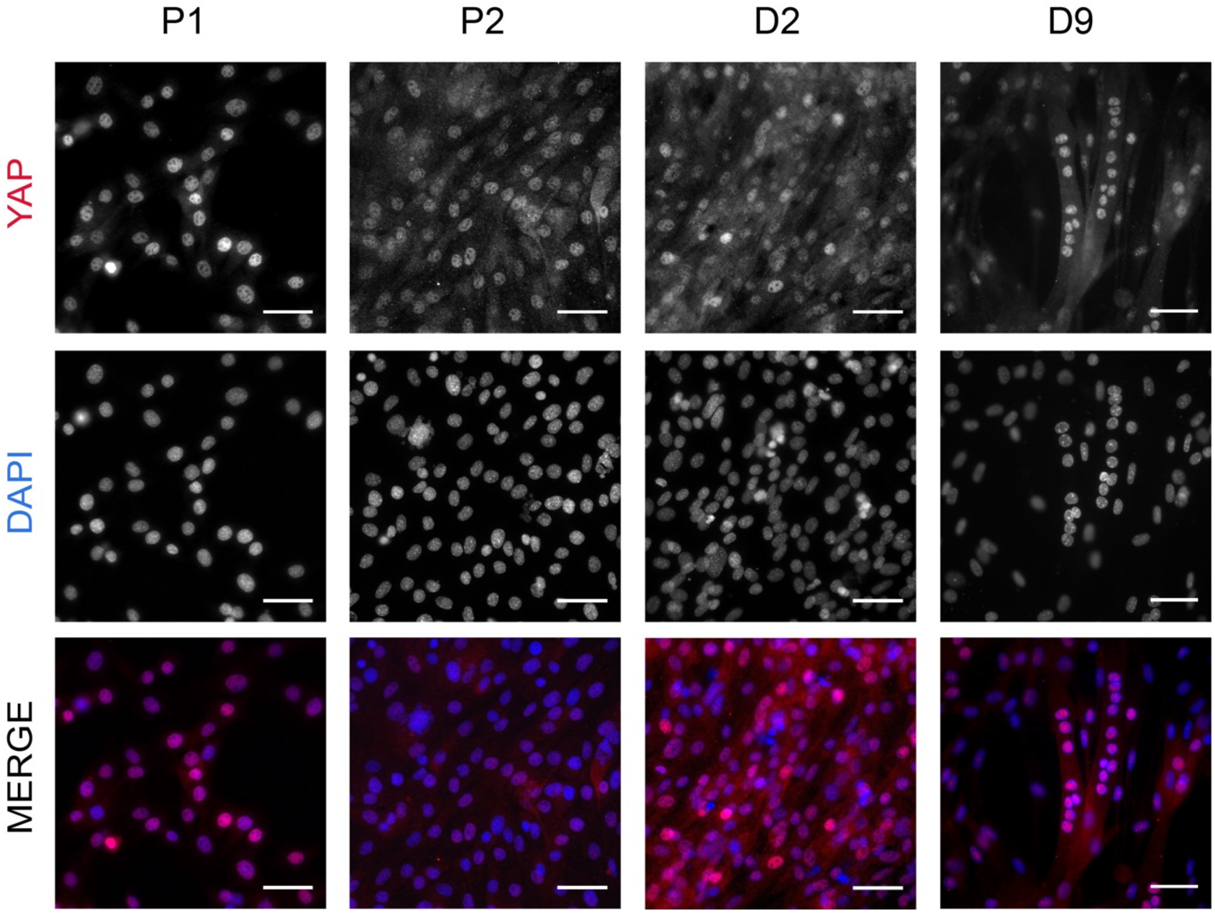

Figure S9 – Epifluorescent images of C2C12 cells stained for YAP and DAPI at P1, P2, D2 and D9. The scale bars are 100 μm.
